# Granzyme A–producing T helper cells are critical for acute graft-versus-host disease

**DOI:** 10.1172/jci.insight.124465

**Published:** 2020-09-17

**Authors:** Sungtae Park, Brad Griesenauer, Hua Jiang, Djamilatou Adom, Pegah Mehrpouya-Bahrami, Srishti Chakravorty, Majid Kazemian, Tanbeena Imam, Rajneesh Srivastava, Tristan A. Hayes, Julian Pardo, Sarath Chandra Janga, Sophie Paczesny, Mark H. Kaplan, Matthew R. Olson

**Affiliations:** 1 Department of Biological Sciences, Purdue University, West Lafayette, Indiana, USA.; 2 Department of Pediatrics and Herman B Wells Center for Pediatric Research and; 3 Department of Microbiology and Immunology, Indiana University School of Medicine, Indianapolis, Indiana, USA.; 4 Departments of Biochemistry and Computer Science, Purdue University, West Lafayette, Indiana, USA.; 5 Department of Biohealth Informatics, School of Informatics and Computing, Indiana University-Purdue University, Indianapolis, Indianapolis, Indiana, USA.; 6 Biomedical Research Centre of Aragon (CIBA), Department of Microbiology, Preventative Medicine and Public Health, Nanoscience Institute of Aragon (INA), Aragon I+D Foundation, IIS Aragon/University of Zaragoza, Zaragoza, Spain.

**Keywords:** Immunology, Inflammation, T cells, Th1 response

## Abstract

Acute graft-versus-host disease (aGVHD) can occur after hematopoietic cell transplant in patients undergoing treatment for hematological malignancies or inborn errors. Although CD4^+^ T helper (Th) cells play a major role in aGVHD, the mechanisms by which they contribute, particularly within the intestines, have remained elusive. We have identified a potentially novel subset of Th cells that accumulated in the intestines and produced the serine protease granzyme A (GrA). GrA^+^ Th cells were distinct from other Th lineages and exhibited a noncytolytic phenotype. In vitro, GrA^+^ Th cells differentiated in the presence of IL-4, IL-6, and IL-21 and were transcriptionally unique from cells cultured with either IL-4 or the IL-6/IL-21 combination alone. In vivo, both STAT3 and STAT6 were required for GrA^+^ Th cell differentiation and played roles in maintenance of the lineage identity. Importantly, GrA^+^ Th cells promoted aGVHD-associated morbidity and mortality and contributed to crypt destruction within intestines but were not required for the beneficial graft-versus-leukemia effect. Our data indicate that GrA^+^ Th cells represent a distinct Th subset and are critical mediators of aGVHD.

## Abstract 2

A subset of granzyme A–producing CD4^+^ T cells accumulate in the intestines of mice and mediate the development of graft-versus-host disease after hematopoietic cell transplantation.

## Introduction

Acute graft-versus-host disease (aGVHD) is a deleterious alloimmune-mediated response that occurs in patients undergoing hematopoietic cell transplant (HCT) for treatment of hematopoietic malignancies or inborn errors. CD4^+^ T helper (Th) cells within the donor graft that recognize allogeneic HLA molecules are major effectors in aGVHD. During aGVHD, Th cells traffic to multiple organs, including the intestines, which is associated with worsened clinical prognosis and decreased ability to treat disease with steroids (1, 2).

Intestinal aGVHD has been described as largely a Th1-mediated response (3–5). However, the role of IFN-γ in aGVHD remains highly controversial. Neither donor- nor host-derived IFN-γ was required for aGVHD (6–8). Instead, IFN-γ played a protective role in aGVHD (9, 10), suggesting that other Th lineages may promote disease.

In this study, we identified a potentially novel subset of granzyme A–producing (GrA-producing) CD4^+^ T cells that were enriched within the intestines of mice with aGVHD and exhibited a phenotype distinct from other Th cells and cytotoxic lymphocytes. Further, GrA^+^ Th cells required STAT3 and STAT6 for their development and lineage identity in vitro and in vivo. We showed that Th-derived GrA contributed to intestinal damage and lethality in multiple murine models of aGVHD. Importantly, GrA^+^ Th cells were dispensable for the beneficial graft-versus-leukemia effect, indicating that GrA^+^ Th cells may represent a novel therapeutic target for treatment of aGVHD.

## Results

### Intestinal GrA^+^ Th cells in aGVHD represent a distinct Th lineage.

**Figure 1:**
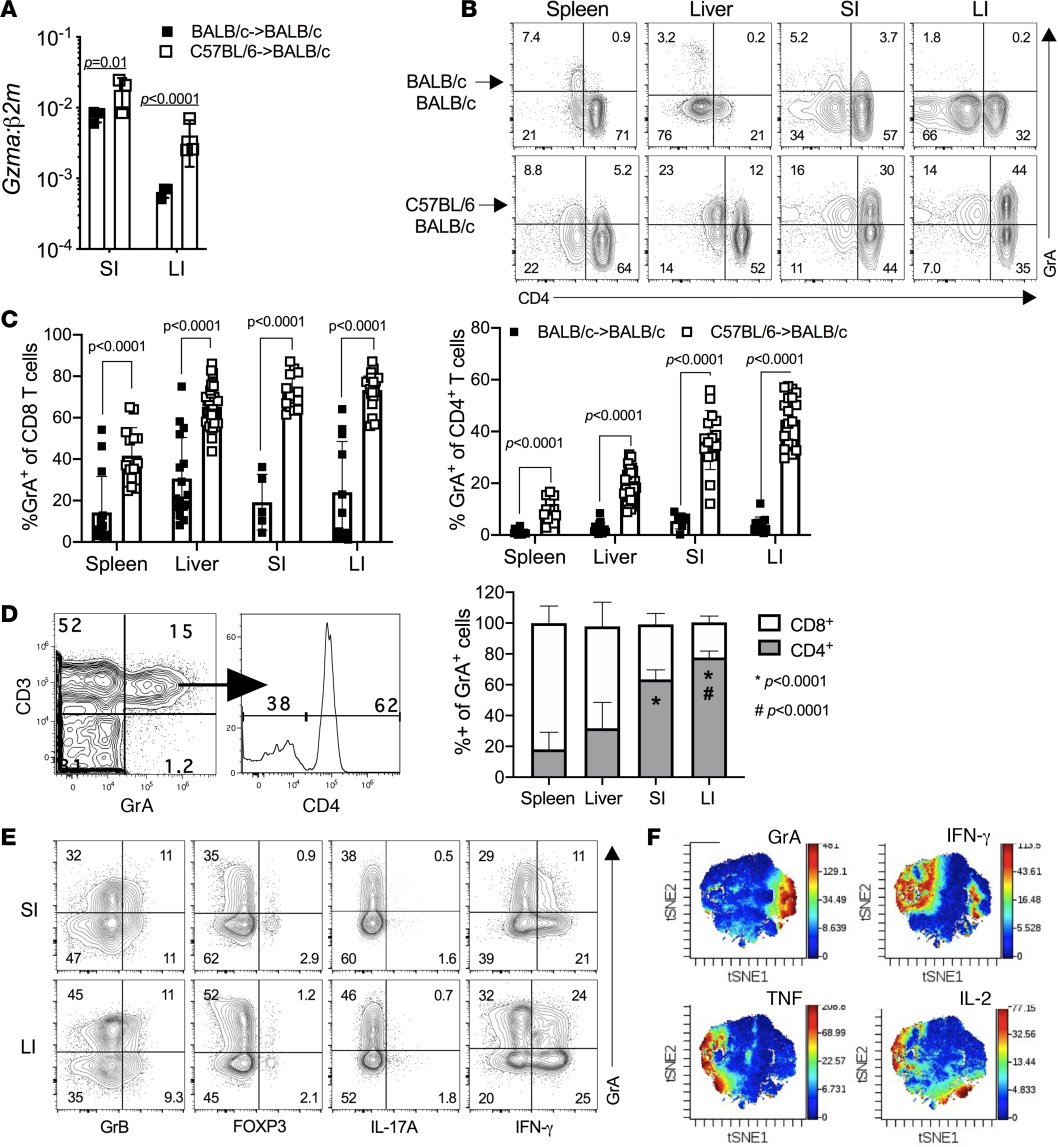
GrA^+^ Th cells are a hallmark of intestinal GVHD and represent a distinct Th subset. Irradiated BALB/c mice received syngeneic (BALB/c**→**BALB/c) or allogeneic (C57BL/6**→**BALB/c) bone marrow and T cells. After transplant (day 10), small intestine (SI) and large intestine (LI) tissue were collected from 3 animals per group for RNA analysis (**A**), or spleen, liver, SI, and LI were harvested for cellular GrA analysis by flow cytometry (**B**). (**C** and **D**) The frequency of GrA^+^CD8^+^ T cells (CD3^+^CD4^–^) or CD4^+^ T cells (CD3^+^CD4^+^) in each tissue from 5–16 syngeneic mice and 14–33 allogeneic mice. (**D**) Percentages of intestinal GrA^+^ Th cells. Left panel, representative plots of GrA and CD3 staining. Right panel, the frequency of CD4^+^ and CD8^+^ T cells within GrA^+^ cells from various organs. *P < 0.05 (Student’s *t* test) as compared with frequency of cells in spleen. (**E**) GrB and FOXP3 expression (unstimulated) and IL-17A and IFN-γ expression (stimulated) by intestinal Th cells from allogeneic mice. Cellular analysis is representative of 4 experiments with 3 mice per group and error bars represent standard deviation of the mean. (**F**) CyTOF analysis of intestinal Th cells, pooled from 10 mice, at day 10 after allogeneic transplant. t-Distributed stochastic neighbor embedding (t-SNE) dimensionality reduction plots represent expression data from GrA, IFN-γ, TNF, and IL-2 staining.

Although the cellular sources were not defined, enhanced *Gzma* expression was previously observed in the intestines during aGVHD but only minimally in the skin and lymphoid organs (11). At 10 days post-HCT, we observed significantly increased expression of *Gzma* in both the small intestine (SI) and large intestine (LI) of mice that received allogeneic bone marrow and T cells as compared with syngeneic controls (*P* = 0.01 and *P* < 0.0001, respectively, Figure 1A). We observed a population of GrA^+^CD8^+^ T cells in all organs examined in allogeneic recipients that was present in lower percentages in syngeneic recipients (Figure 1, B and C). Unexpectedly, CD4^+^ T cells also produced GrA in allogeneic recipients and were the dominant population of GrA-producing T cells within the small and large intestines, the latter being where CD4^+^ T cells accounted for approximately 75% of the GrA-producing cells (Figure 1, B–D).

A limited proportion of intestinal GrA^+^ Th cells coexpressed GrB in the SI or LI, indicating that these cells did not acquire a classic cytotoxic phenotype (Figure 1E) (12–15). GrA^+^ cells did not coexpress FOXP3 or IL-17A (Figure 1E) but exhibited partial coexpression of IFN-γ, which was slightly more pronounced in the LI versus the SI (~40% vs. ~30%, Figure 1E). To determine if GrA^+^ Th cells were related to IFN-γ–producing Th1 cells, we analyzed lineage-specific cytokine and transcription factor expression (Supplemental Table 2; supplemental material available online with this article; https://doi.org/10.1172/jci.insight.124465DS1) within intestinal CD3^+^CD4^+^ T cells using time-of-flight cytometry (CyTOF). GrA^+^ Th cells were spatially clustered, indicating that GrA expression is limited to a distinct population of Th cells and is not shared by multiple Th subsets (Figure 1F). In contrast, IFN-γ^+^ Th cells were distributed between 2 major populations, those that spatially segregated with TNF^+^IL-2^+^ cells (i.e., prototypic Th1 cells) and those that partially overlapped with GrA^+^ Th cells (Figure 1F). Cells that expressed high levels of GrA exhibited minimal overlap with other lineage-specific cytokines, many of which were expressed in low amounts (Supplemental Figure 1). These data indicate that GrA^+^ Th cells are not typical Th1 cells and may represent a novel Th cell type that responds to signals involved with intestinal damage or inflammation.

### GrA is expressed by human Th cells in a PBMC-induced model of GVHD.

**Figure 2:**
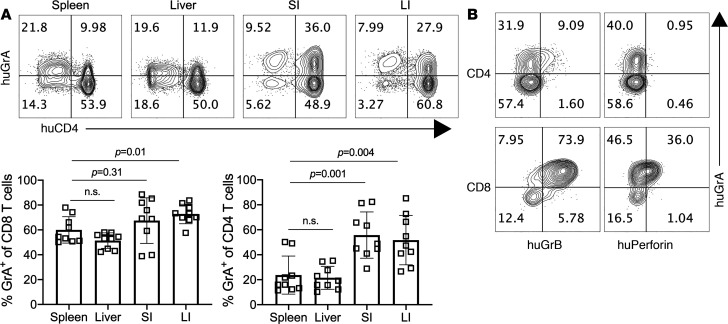
GrA^+^ Th cells are prevalent in the intestines and display a noncytolytic phenotype in a human PBMC-driven xenograft model of GVHD. (**A**) Representative FACS plots depicting GrA expression by CD3-gated human CD4^+^ and CD4^–^ (i.e., CD8^+^) T cells and quantitation of GrA^+^CD4^+^ and CD8^+^ T cells in each organ. (**B**) Representative FACS plots depicting human GrA, GrB, and perforin staining of gated CD3^+^CD4^+^ or CD3^+^CD8^+^ T cells. Error bars represent standard deviation of the mean. Human PBMC NRG mouse experiments represent 9 NRG mice that received PBMCs from 2 distinct donors. Statistical significance was determined by 1-way ANOVA with a Tukey’s posttest for multiple comparisons.

Our data indicate that CD4^+^ Th cells are an important source of GrA and thus may be relevant to human GVHD. To examine this further, we adopted an established model (16) of human PBMC-induced GVHD in immune-compromised NRG mice. Briefly, human PBMCs from 2 individual donors were injected into NRG mice, and mice were monitored until they reached 20% body weight loss or a significant clinical score (>3). At this endpoint, mice were euthanized, and immune cells were isolated from the spleen, liver, SI, and LI and analyzed for human GrA^+^ Th cells by flow cytometry. Parallel to our mouse studies, GrA-producing human Th cells were elevated within the intestines, where they were the dominant cellular source of GrA. In contrast, GrA-producing CD8^+^ T cells were uniform across the organs examined and were a smaller component of the GrA^+^ cells in the intestines (Figure 2A). Human GrA-producing Th cells did not express perforin, and only a small proportion expressed modest GrB, while GrA^+^CD8^+^ T cells predominantly coexpressed GrB and perforin (Figure 2B), suggesting that GrA-producing Th cells were distinct from cytolytic CD8^+^ T cells and had a noncytolytic phenotype. These data provide further evidence that human Th cells are capable of producing GrA within the context of GVHD and potentially contribute to GVHD pathogenesis in humans.

### GrA^+^ Th cell differentiation in vitro.

**Figure 3:**
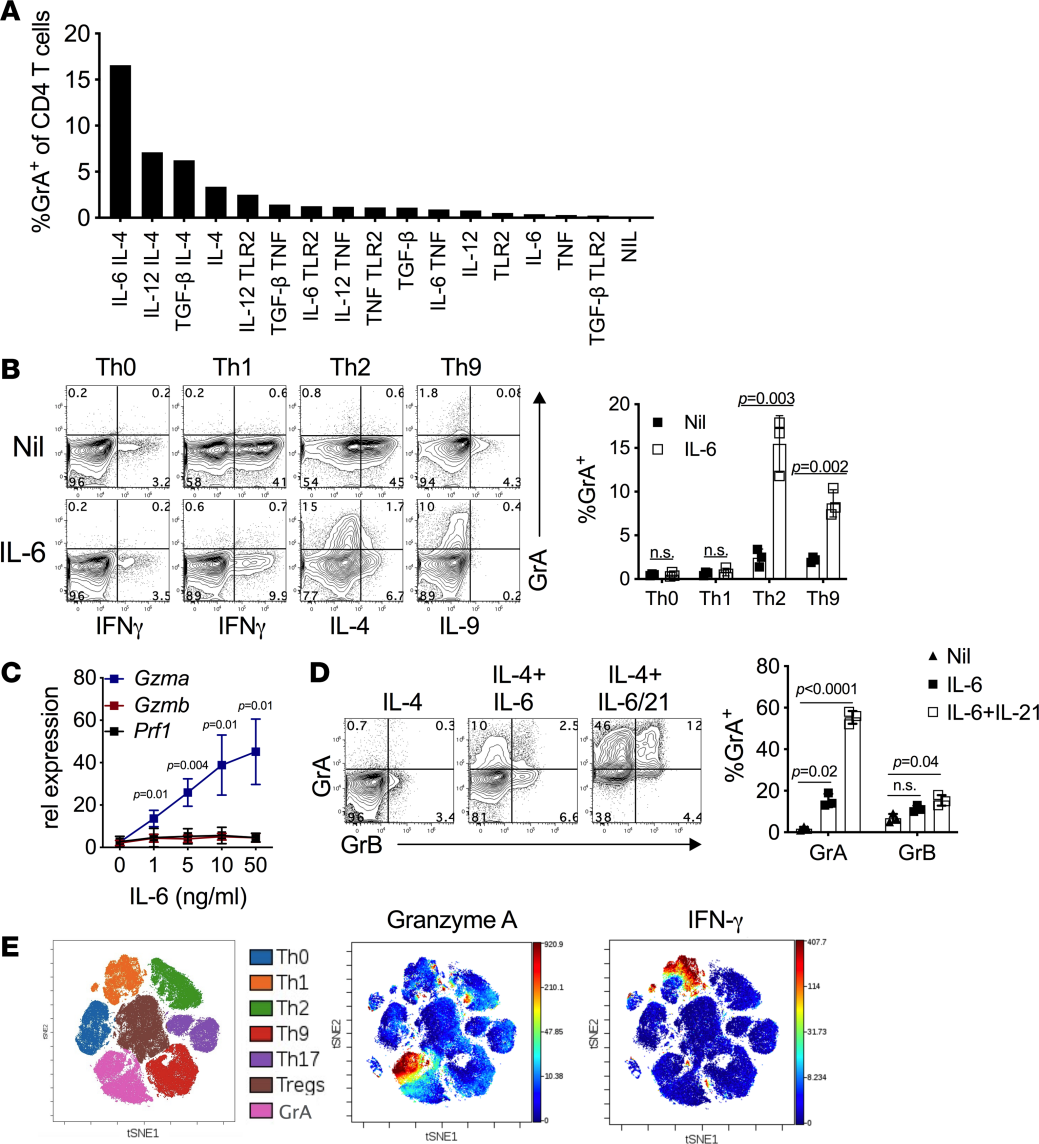
IL-4 and IL-6 signaling synergize in the induction of GrA^+^ Th cells. (**A**) Naive Th cells were differentiated in the presence of the indicated cytokines (TLR2 = PAM3CSK4) and stained for intracellular GrA. Data represents a single screen with cells pooled from 3 mice. These data are representative of 3 individual screening experiments. Nil, no treatment. (**B**) Indicated Th cells from 3 individual mice were cultured with/without IL-6 (10 ng/mL) followed by stimulation and intracellular staining for cytokines and GrA. Representative contour plots are depicted (left panel), and the frequency of GrA^+^ cells is depicted in the right panel. (**C**) Cells from 3 mice were cultured with IL-4, anti–IFN-γ, and increasing doses of IL-6. On day 5 of culture mRNA was measured by real-time PCR. Statistical analysis was done by Student’s *t* test, and reported values are corrected for multiple comparisons. (**D**) Cells from 3 individual mice were cultured with IL-4, IL-4+IL-6, or IL-4+IL-6 and with IL-21 (100 ng/mL) added at day 3 of culture. On day 5 of culture, cells were harvested and stained for intracellular GrA and GrB (left panel), and frequencies of GrA^+^ and GrB^+^ cells were determined by flow cytometry (right panel). Statistical analysis was done by 2-way ANOVA with Holm-Šidák correction for multiple comparisons. (**E**) Indicated Th subsets were analyzed by CyTOF and displayed in a dot overlays plot. Cytokine screening assays were performed 4 times with pooled naive CD4^+^ T cells. Th polarization experiments were repeated 3–6 times. Error bars represent standard deviation of the mean. CyTOF analysis was performed twice with pooled naive CD4^+^ T cells isolated from multiple animals.

**Figure 4:**
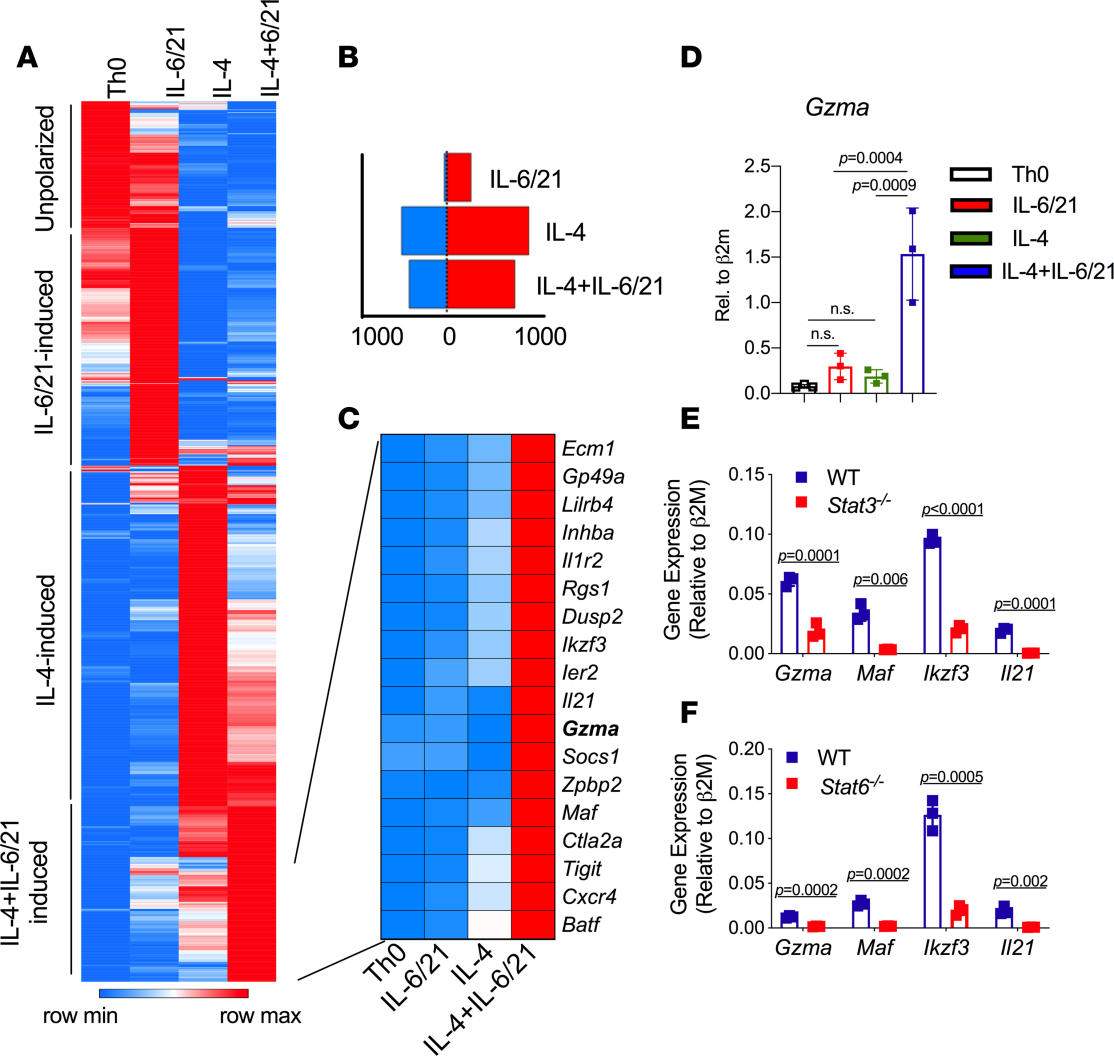
IL-6/21 alters the IL-4–induced transcriptional program and promotes *Gzma* production. (**A**) Heatmap of statistically significant (FDR < 0.05; fold change > 2) gene expression of cells cultured under Th0, Th0+IL-6/21 (IL-6/21), Th0+IL-4 (IL-4), or Th0+IL-4+IL-6/21 (IL-4+IL-6/21) based on a 2-fold change cutoff. Data represent the means expression from cells isolated from 3 animals per condition. (**B**) Number of up- or downregulated genes as compared with Th0 cells. (**C**) Select genes from the subset of genes that are uniquely enriched in cells cultured with IL-4+IL-6/21. (**D**) Verification of *Gzma* expression via real-time PCR. (**E**) mRNA expression levels in cells isolated from C57BL/6 *Stat3*^fl/fl^ *Cd4*-Cre^–^ (WT) and *Stat3*^fl/fl^ *Cd4*-Cre^+^ mice and (**F**) WT and Stat6^–/–^ mice (BALB/c) after culture with IL-4+IL-6/21. Experiments from **E** and **F** are representative of 2 individual experiments with cells isolated from 2–3 mice per experiment. Error bars represent standard deviation of the mean. Statistical significance was determined by Student’s *t* test.

To determine the signals that promote GrA^+^ Th cell differentiation, we screened potential combinations of cytokines that are involved with intestinal homeostasis or inflammation. While many cytokine combinations resulted in low expression of GrA by CD4^+^ T cells (~5% GrA^+^ cells or less), the combination of IL-4 and IL-6 resulted in increased GrA^+^ Th cell differentiation (Figure 3A). Similarly, unpolarized (Th0) and polarized Th1, Th2, and Th9 cells showed minimal GrA protein expression after in vitro differentiation (Figure 3B). However, IL-6 diminished IL-4 (Th2) and IL-9 (Th9) production (17) and enhanced GrA production (Figure 3B). In the context of IL-4, IL-6 induced *Gzma* mRNA in a dose-dependent manner, without the induction of *Gzmb* or *Prf1* (Figure 3C). Addition of IL-21 alone, which enhances polarization of other Th subsets (18), did not induce GrA production by Th cells (data not shown), but significantly enhanced GrA production when used in combination with IL-6 (~58%) as compared with IL-6 alone (~12%), with an increase in GrB (Figure 3D) parallel to that observed ex vivo. Similar to our analysis of intestinal GrA^+^ Th cells, GrA expression was largely limited to cells cultured with IL-4+IL-6/21 and spatially segregated from other polarized Th subsets in parallel CyTOF analysis (Figure 3E). To determine if in vitro–derived GrA^+^ Th cells were also transcriptionally distinct, we performed RNA-Seq analysis from unpolarized (Th0), IL-6/21, IL-4, and IL-4+IL-6/21 polarized cells. IL-6/21 alone induced or repressed the expression of fewer genes (i.e., 252) as compared with cells cultured with IL-4 or IL-4+IL-6/21 (1206 with IL-4, 1001 with IL-4+IL-6/21; Figure 4, A and B). IL-4 and IL-6 induced expression of distinct sets of genes and addition of IL-6/21 to IL-4–cultured cells suppressed a subset of IL-4–induced genes while other IL-4–induced genes were relatively unchanged (Figure 4A, Supplemental Figure 2A, and Supplemental Table 4). Conversely, IL-4 blunted the expression of IL-6/21–induced genes (Figure 4A, Supplemental Figure 2B), suggesting that these cytokine-signaling pathways act in opposition. Interestingly, an additional subset of genes were synergistically induced by IL-4 and IL-6/21 and characterized by high expression of *Gzma* and other factors that contribute to Th cell differentiation (i.e., *Ikzf3*, *Maf*, *Batf*; Figure 4, A, C, and D; Supplemental Figure 2, C and D; and Supplemental Table 4). These data indicate that IL-6/21 signaling redirects cells from an IL-4–driven Th2 phenotype into a GrA-producing lineage by repressing specific aspects of the Th2 lineage and inducing a novel pattern of gene expression. However, adoptive transfer of Th cells polarized with IL-4 alone or with IL-4 and IL-6/21 was poor at inducing aGVHD. Both sets of recipient mice exhibited similar low clinical scores, weight loss, colon lengths, and lack of mortality after HCT (*P* = 0.41, Supplemental Figure 3, A–C). The poor pathogenicity of these cells is likely attributed to a loss of GrA production and large gains in IL-4 production after HCT (Supplemental Figure 3D).

Because IL-4 and IL-6/21 respectively activate STAT6 and STAT3, we examined targeted mRNA levels in IL-4+IL-6/21–cultured cells isolated from WT, *Stat6*^–/–^, and *Stat3*^–/–^ (*Stat3*^fl/fl^ ^CD4-Cre^) mice by real-time PCR. Elevated expression of *Gzma*, *Maf*, *Ikzf3*, and *Il21* in IL-4+IL-6/21–cultured cells required both STAT6 and STAT3 as cells from deficient mice exhibited significantly reduced (*P* < 0.006) expression of all these genes (Figure 4, E and F). These data are consistent with our RNA-Seq analysis and demonstrate a necessity of both STAT6 and STAT3 in activating *Gzma* expression and the associated gene expression profile. Together, these data indicate that IL-4/STAT6 and IL-6/IL-21/STAT3 signals synergistically drive the GrA^+^ Th cell gene signature.

**Figure 5:**
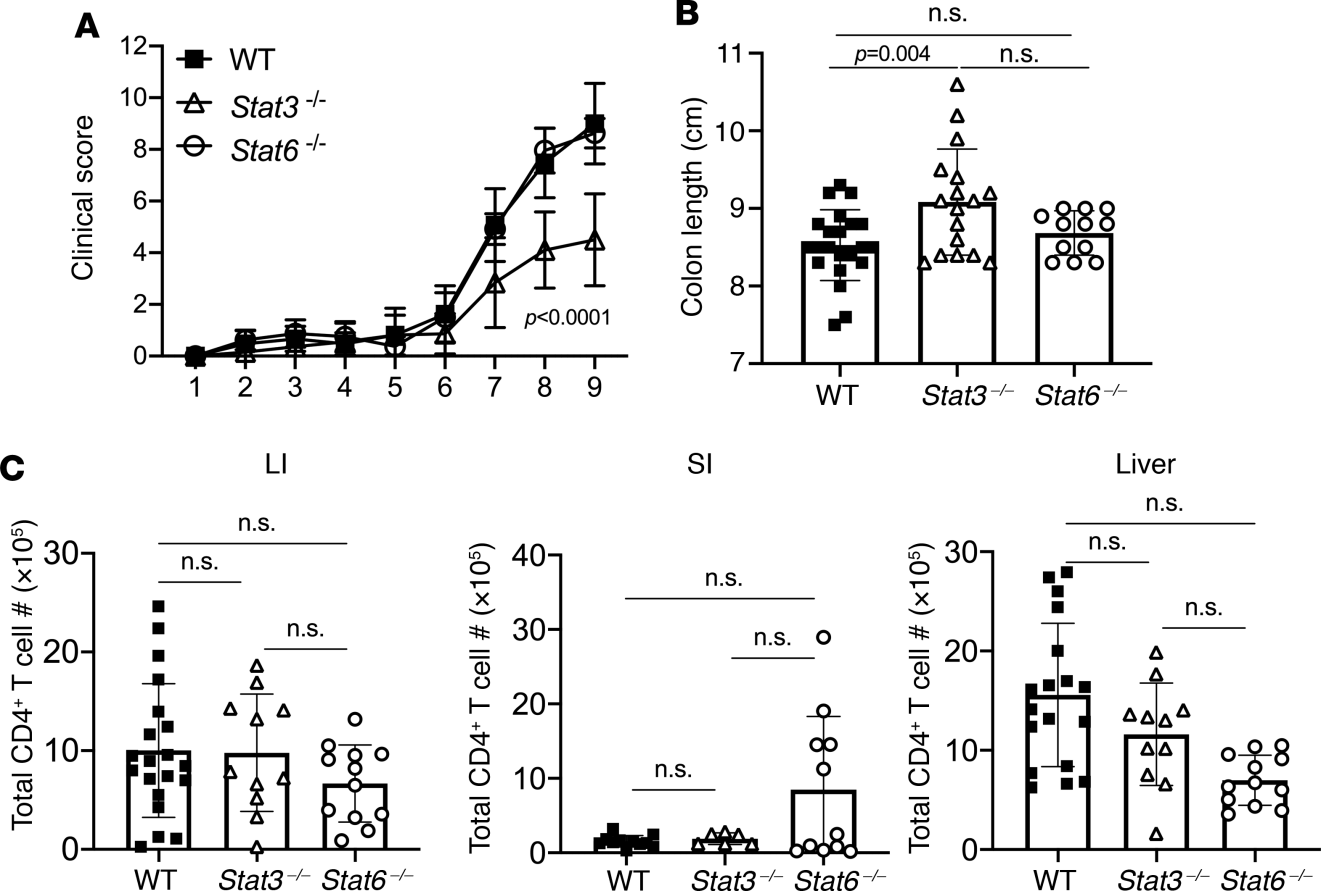
STAT3- and STAT6-deficient Th cells differentially drive aGVHD. Lethally irradiated BALB/c mice received C57BL/6-derived bone marrow and WT CD8^+^ T cells with CD4^+^ T cells from WT (*n* = 21), *Stat3*^fl/fl^ CD4-Cre^+^ (*Stat3*^–/–^) (*n* = 19), or *Stat6*^–/–^ mice (*n* = 12). Clinical scores (**A**) and colon length (**B**) of recipient mice harvested at day 9 after HCT. (**C**) Total viable CD4^+^ T cell numbers from the LI, SI, and liver. Data are pooled from 3 individual experiments where error bars represent standard deviation of the mean. n.s., not significant, *P* > 0.05 (1-way ANOVA with Tukey’s posttest for multiple comparisons).

**Figure 6:**
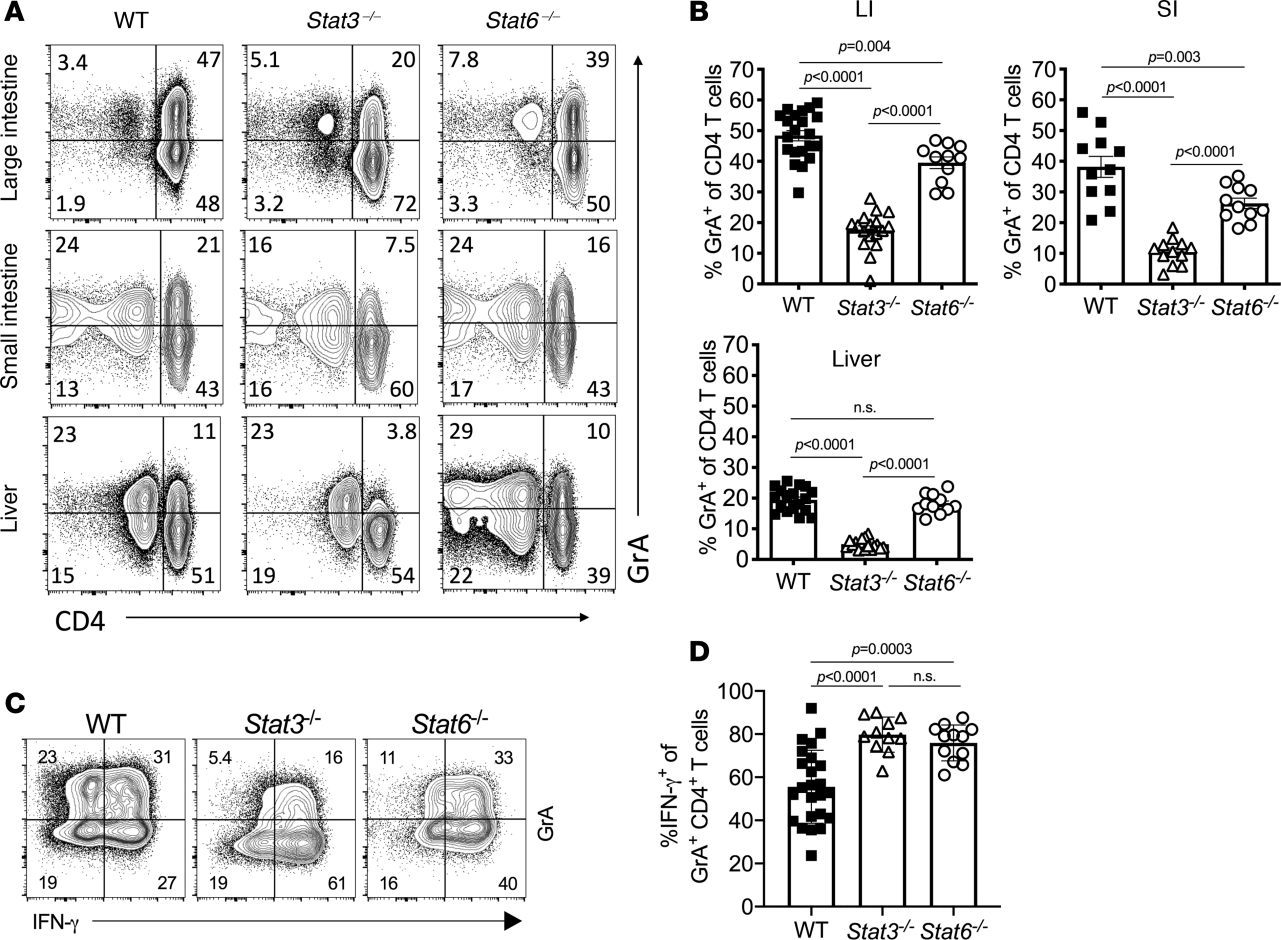
STAT3 and STAT6 induce GrA^+^ Th cells’ differentiation and maintain lineage identity during GVHD. T cells isolated from mice from Figure 5 were analyzed for intracellular expression of GrA. (**A**) Representative FACS plots of intracellular GrA and CD4 expression from CD3^+^ cells isolated from LI, SI, and liver. (**B**) Quantification of the frequency of GrA^+^CD4^+^ T cells from the LI, SI, and liver. (**C**) Representative FACS plots of GrA and IFN-γ from CD4^+^ T cells isolated from LI and stimulated with PMA and ionomycin. (**D**) Quantification of the frequency of GrA^+^CD4^+^ T cells expressing IFN-γ. Data are pooled from 3 individual experiments where error bars represent standard deviation of the mean. Statistical significance was determined by 1-way ANOVA with Tukey’s posttest for multiple comparisons.

As we identified both STAT3 and STAT6 to be critical for GrA^+^ Th cell differentiation in vitro, we asked whether these signals were also required for their differentiation during aGVHD. In these studies, lethally irradiated BALB/c mice received T cell–depleted bone marrow and CD8^+^ T cells from WT C57BL/6 mice and purified CD4^+^ T cells from WT, *Stat3*^–/–^, or *Stat6*^–/–^ mice. After HCT, mice that received WT and *Stat6*^–/–^ CD4^+^ T cells exhibited similar increased clinical scores, but these were significantly reduced in *Stat3*^–/–^ CD4^+^ T cell recipients (*P* < 0.0001) and accompanied by increased colon length (*P* = 0.004) as compared with WT controls (Figure 5, A and B). Mice that received either *Stat3*- or *Stat6*-deficient CD4^+^ T cells had similar CD4^+^ T cell numbers in the LI, SI, and liver as compared with WT recipients, suggesting that neither factor was required for the survival or expansion of Th cells at this time point (Figure 5C). While both *Stat3*- and *Stat6*-deficient CD4^+^ T cells exhibited decreased GrA expression in the LI and SI, this was much more dramatic in *Stat3*-deficient Th cells (*P* < 0.0001 vs. *P* = 0.004 or *P* = 0.003) and also resulted in reduced GrA expression in the liver, where *Stat6*-deficient cells had similar GrA expression to WT cells in this organ (*P* = 0.33, Figure 6, A and B). Interestingly, while approximately 50% of LI-derived GrA^+^CD4^+^ T cells expressed IFN-γ in these experiments, this was significantly increased (~80%) in *Stat3*- and *Stat6*-deficient CD4^+^ T cells (Figure 6, C and D; *P* < 0.0001 and *P* = 0.0003, respectively), indicating that both STAT3 and STAT6 play critical roles in suppressing a Th1-like phenotype in these cells and aid in maintaining the GrA^+^ Th cell lineage identity.

### Th-derived GrA is required for aGVHD.

**Figure 7:**
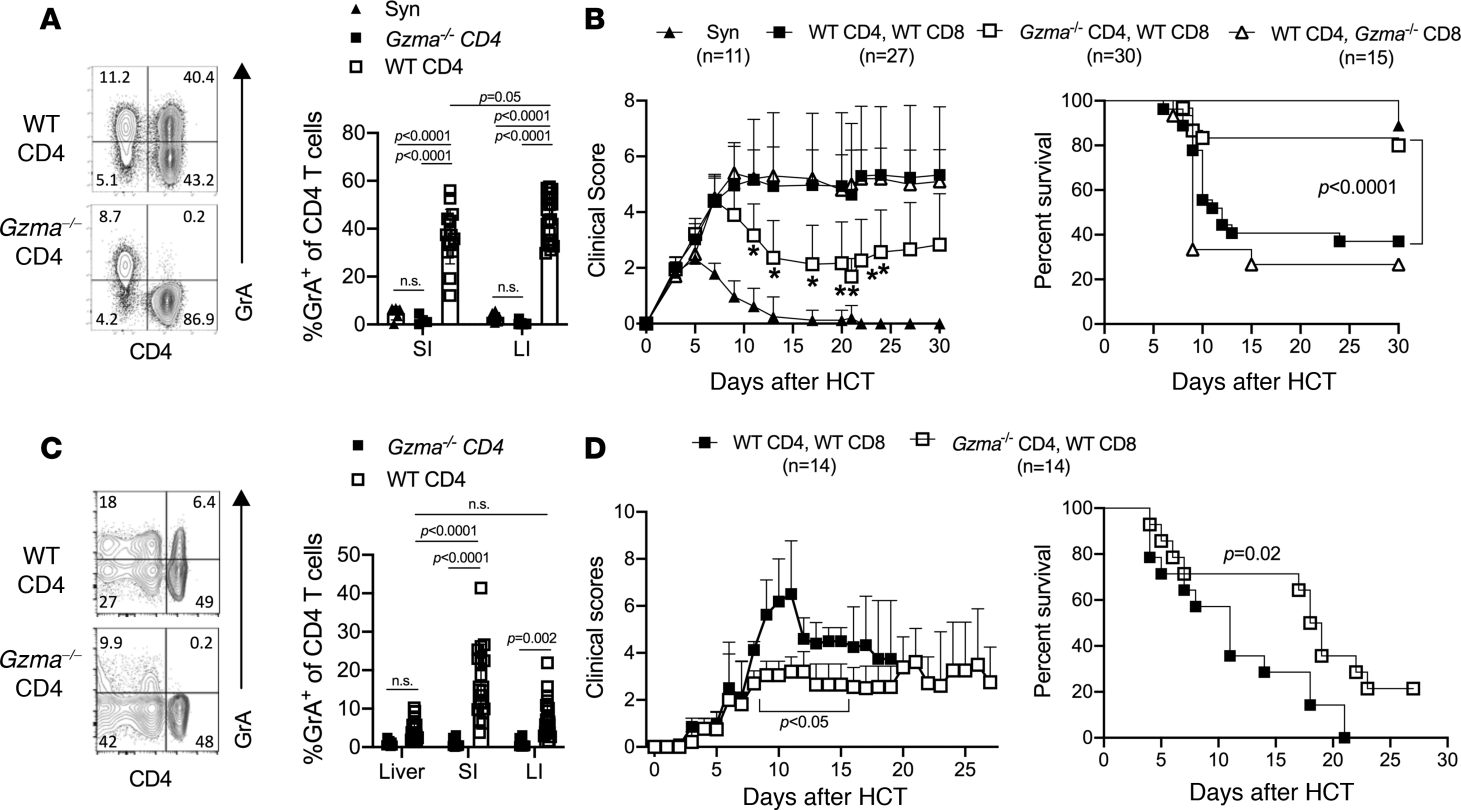
Th-derived GrA contributes to immunopathology during aGVHD. (**A**) BALB/c mice received syngeneic (Syn, *n* = 11) or C57BL/6 bone marrow with purified CD8^+^ and CD4^+^ T cells from WT or *Gzma*^–/–^ C57BL/6 mice (WT CD4^+^/WT CD8^+^, *n* = 27; *Gzma*^–/–^ CD4^+^/WT CD8^+^, *n* = 30; WT CD4^+^/*Gzma*^–/–^ CD8^+^, *n* = 15). LI cells were harvested (at day 10 post-HCT) and assessed for GrA production by CD3^+^ cells by flow cytometry. Representative plots are gated on CD3^+^ cells (left panel), and the frequency of GrA^+^CD3^+^CD4^+^ T cells was quantified (right panel) based on the flow cytometry plots. Clinical scores and survival curves (**B**) from BALB/c recipient mice after HCT as per panel **A**. C3.SW-H2^b^/SnJ mice received bone marrow and T cells as per panel **A**. (**C**) Representative contour plots depict GrA^+^ T cells from the LI and the frequencies of GrA^+^CD4^+^ T cells in recipient mice isolated from the liver, SI, and LI. (**D**) Clinical scores and survival plot of C3.SW-H2^b^/SnJ that received WT (*n* = 14) or *Gzma*^–/–^ CD4^+^ T cells (*n* = 14). Clinical score and survival plots represent pooled data across multiple experiments with mouse numbers indicated. Error bars represent standard deviation of the mean. *P < 0.05 by Student’s *t* test in **B** and **D**. Statistical significance in survival plots was performed by log-rank test (see Methods). One-way ANOVA with Tukey’s posttest was used to determine statistical significance in **A** and **C**.

Having identified a population of intestinal GrA^+^ Th cells in mice with aGVHD, we next questioned whether Th-derived GrA was important for development of disease. In these experiments, we used the C57BL/6 into BALB/c model of aGVHD because pathology in this system is dependent on CD4^+^ T cells (19). Briefly, mice received syngeneic or allogeneic cells as above, and additional groups of mice received allogeneic bone marrow and combinations of CD4^+^ and CD8^+^ T cells from WT or *Gzma*^–/–^ animals. As expected, mice that received *Gzma*^–/–^ CD4^+^ T cells had normal percentages of GrA^+^CD8^+^ T cells but lacked GrA^+^ Th cells (Figure 7A and data not shown). Further, *Gzma*^–/–^ Th cell recipients had similar intestinal total cell numbers, inflammatory cell numbers, frequencies of donor-derived (i.e., H-2K^b+^) Th cells, and frequencies of IFN-γ^+^ and IL-17A^+^ Th cells as compared with controls (Supplemental Figure 4, A–C; and data not shown). These data indicate equivalent proliferation, trafficking, and activation of WT and GrA-deficient Th cells in the intestines. In other analyses, mice that received WT Th cells exhibited significantly increased clinical scores and mortality by day 10 post-HCT as compared with syngeneic controls (*P* < 0.05, Figure 7B). Mice that received *Gzma*^–/–^ Th cells exhibited significantly reduced clinical scores (*P* = 0.02–0.05) and mortality (*P* < 0.0001, Figure 7B) as compared with mice that received WT Th cells. Surviving *Gzma*^–/–^ Th recipient mice persisted until 50 days post-HCT (Supplemental Figure 5, A and B). Mice that received *Gzma*^–/–^ CD8^+^ T cells, but WT CD4^+^ T cells, exhibited similar clinical scores and mortality to mice that received WT CD4^+^ and CD8^+^ T cells (Figure 7B). These data indicate that CD4^+^ T cell–derived, but not CD8^+^ T cell–derived, GrA is critical for the progression of aGVHD in this CD4^+^ T cell–dependent model. As a whole, GrA^+^ Th cells contribute to disease without altering the recruitment of inflammatory cells into the intestines or altering the ability of Th cells to produce IFN-γ, IL-4, or IL-17A.

Because only a fraction of HCTs performed clinically are from MHC major mismatched donors, we aimed to confirm the role of GrA^+^ Th cells in a minor histocompatibility (miHA) mismatch model of GVHD. To this end, we induced GVHD in lethally irradiated C3.SW-H2^b^/SnJ mice by HCT with bone marrow and T cells from C57BL/6 mice as per above. Similar to the allogeneic model, GrA^+^CD4^+^ T cells were significantly increased in the SI (*P* < 0.0001) in comparison with the liver in this model (Figure 7C). GrA^+^ Th cells from the SI of these animals also displayed a similar cytokine profile as observed in major mismatch recipients (Supplemental Figure 4D). Importantly, *Gzma*^–/–^ Th recipients exhibited reduced clinical scores (days 8–15, *P* < 0.05) and enhanced survival as compared with WT recipients (*P* = 0.02, Figure 7D). Additionally, lack of GrA production by CD4^+^ T cells in these models had no effect on total LI infiltrating cells, total number of CD4^+^ T cells in the LI, numbers of inflammatory cells, or Th lineage-associated cytokine expression (Supplemental Figure 4, E–G). Interestingly, the role of GrA^+^ Th cells in promoting aGVHD in the miHA-driven model was more modest than that observed in the major mismatch model of aGVHD. This may be due to a greater dependency of CD8^+^ T cells in the miHA-induced model as compared with the more CD4^+^ T cell–dependent nature of the major mismatch model (19). Regardless, these data indicate that GrA^+^ Th cells contribute to disease severity in both MHC major mismatch– and miHA-driven aGVHD without altering immune cell infiltration or effector function.

### CD4^+^ T cell–derived GrA is dispensable for the beneficial graft-versus-leukemia response.

HCT is an effective therapy for treatment of hematological malignancies where allo- or miHA-specific T cells from the donor aid in suppression of tumor cell growth. We demonstrated above that GrA^+^ Th cells play a pathogenic role in driving aGVHD, but whether they are required for the beneficial graft-versus-leukemia (GVL) response is unknown. To address this, we performed mixed GVHD/GVL experiments where irradiated BALB/c mice received syngeneic or allogeneic HCT with T cells from WT, *Gzma*^–/–^, or *Gzma*^–/–^ CD4^+^ T cells and WT CD8^+^ T cells along with GFP^+^ MLL-AF9 leukemia cells. Given that MLL-AF9 cells express MHC-II after transfer and are recognized by both CD4^+^ and CD8^+^ T cells in vitro and in vivo (20, 21), we used this model to assess the relative contributions of GrA produced by all T cells (i.e., CD4^+^ and CD8^+^ T cells) versus that produced only by CD4^+^ T cells in both the GVHD and GVL response.

**Figure 8:**
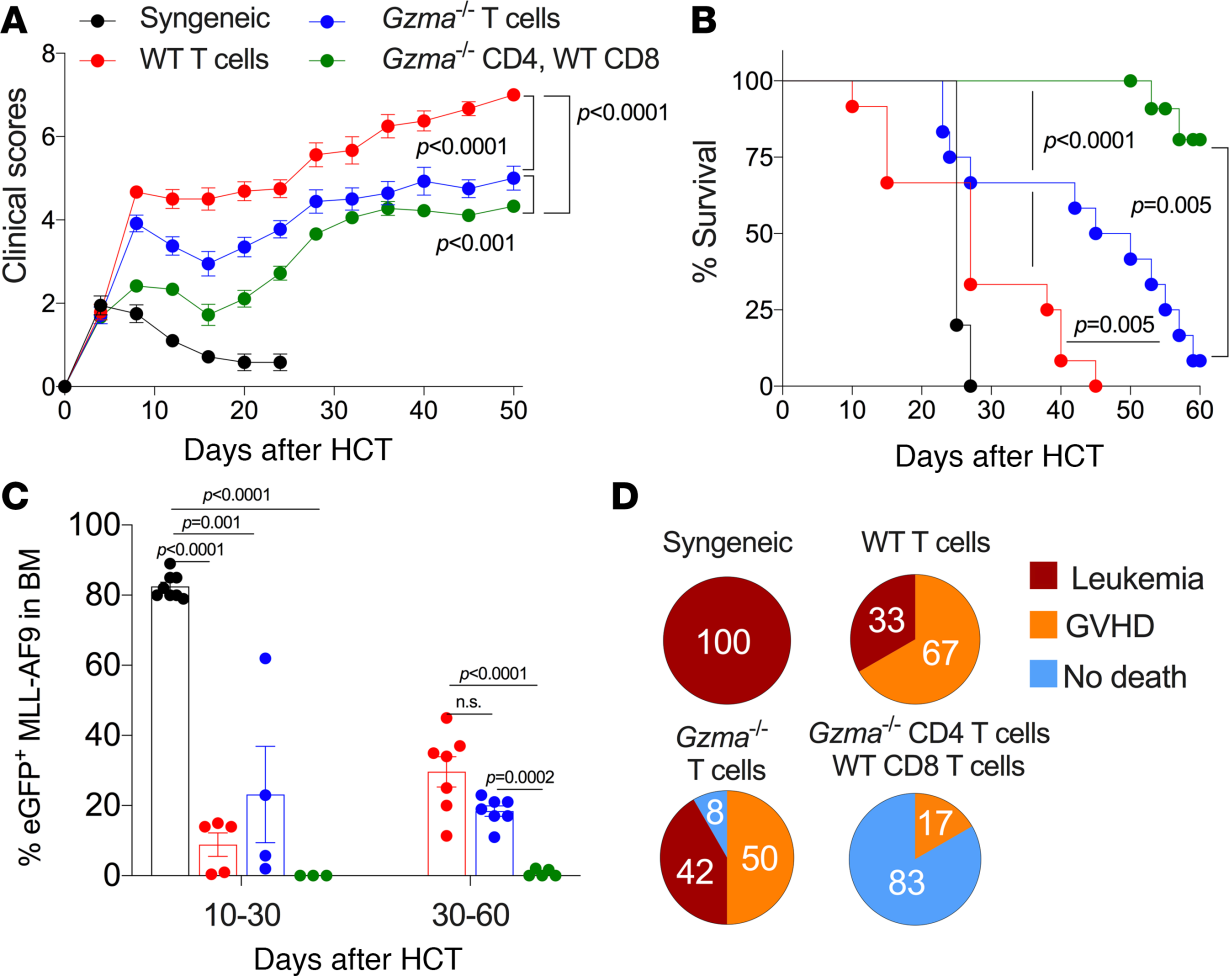
Th cell–derived GrA is not required for the beneficial GVL effect of HCT. Lethally irradiated BALB/c mice received 10^4^ GFP^+^ MLL-AF9 leukemia cells, bone marrow, and T cells from syngeneic controls or allo-HCTs from C57BL/6 mice with WT T cells, *Gzma*^–/–^ T cells, or *Gzma*^–/–^ CD4^+^ T cells and WT CD8^+^ T cells. After transplant, mice were monitored for clinical score (**A**) and mortality (**B**). (**C**) At a predetermined clinical endpoint (or selected as controls if mice did not reach the clinical endpoint), mice were euthanized, and the frequency of GFP^+^ MLL-AF9 cells was quantified in the bone marrow by flow cytometry. The percentages of mice that succumbed to leukemia or GVHD (**D**) were determined based on clinical score and the frequency of GFP^+^ MLL-AF9 cells present in the bone marrow as per panels **A** and **C**. Error bars represent standard error of the mean in panel **A** from 12 mice per group and the standard deviation in panel **C** from the number of mice indicated. Statistical difference in survival plots was performed by log-rank test (see Methods) and for frequency of GFP^+^ cells by 2-way ANOVA with Holm-Šidák correction for multiple comparisons.

After syngeneic HCT, despite minimal development of clinical score, we observed rapid death of 100% of these mice due to high GFP^+^ leukemia burden in the bone marrow (Figure 8, A–D). In contrast, mice with WT allo-HCT rapidly developed clinical scores and had a similar mortality rate as that of the syngeneic controls; however, the majority of deaths occurred from GVHD (67%) rather than leukemia (33%) (i.e., high clinical scores, lower GFP^+^ tumor burden; Figure 8, A–D). Allogeneic HCT with *Gzma*^–/–^ T cells resulted in reduced GVHD-associated clinical scores and mortality as compared with WT controls (*P* < 0.0001 and *P* = 0.005, respectively). Additionally, *Gzma*^–/–^ T cell recipients had similar frequencies of GFP^+^ tumor cells in the bone marrow (*P* > 0.05) and only modestly increased mortality due to leukemia (42%, *P* > 0.05 as compared with WT allo-HCT recipients; Figure 8, A–D). Interestingly, mice receiving an allo-HCT with *Gzma*^–/–^ CD4^+^ T cells and WT CD8^+^ T cells exhibited the lowest clinical scores (*P* < 0.001 vs. *Gzma*^–/–^ T cells and *P* < 0.0001 vs. WT T cells) and mortality as compared with all other allo-HCT recipients (*P* < 0.0001 vs. *Gzma*^–/–^ T cells and *P* < 0.0001 vs. WT T cells). Further, *Gzma*^–/–^ CD4^+^ T cell recipients exhibited no signs of GFP^+^ leukemia cells in the bone marrow upon euthanization, and the only cause of death was GVHD (17%, Figure 8, A–D). Intriguingly, GrA-deficient Treg cells have reduced capacity to regulate allo-specific T cell responses during GVHD (22), and this reduced Treg function may explain the enhanced tumor cell clearance we observed here. Together, these data suggest that CD4^+^ and CD8^+^ T cell–derived GrA have different effects on the outcomes of GVHD and GVL and that CD4^+^ T cell–derived GrA is dispensable for GVL.

### GrA^+^ Th cells are associated with intestinal crypt damage.

**Figure 9:**
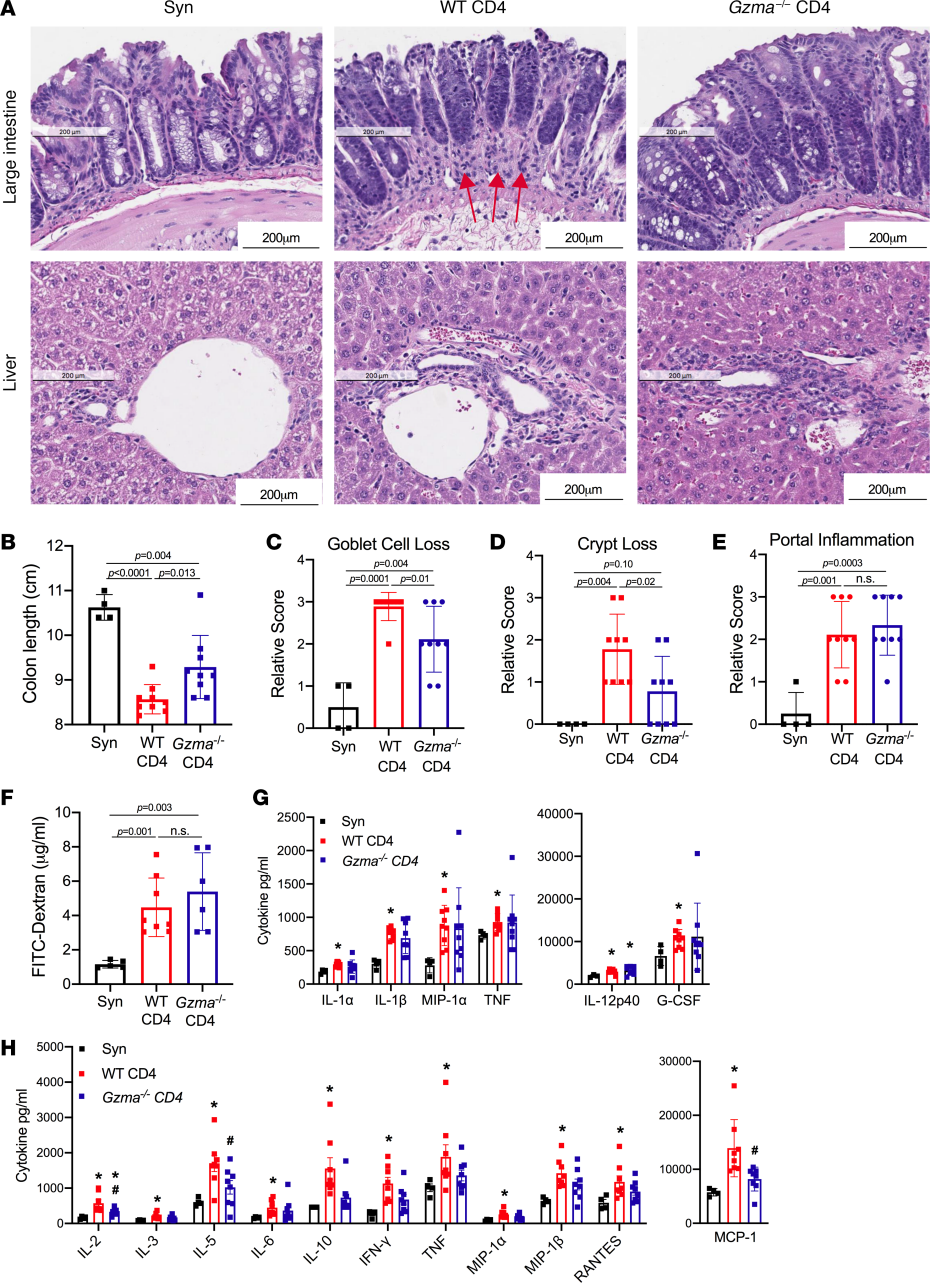
CD4^+^ T cell–derived GrA induces intestinal crypt damage. (**A**) H&E staining of LI and liver tissue from BALB/c mice that received syngeneic (Syn) bone marrow and T cells or from C57BL/6 mice with WT CD8^+^ T cells and CD4^+^ T cells from WT or *Gzma*^–/–^ mice at 9 days post-HCT. (**B**) Colon lengths from mice in panel **A**. Quantification of goblet cell loss (**C**), crypt loss (**D**), and liver portal inflammation (**E**). (**F**) FITC-dextran concentrations in serum after oral gavage of BALB/c recipients at day 9 after HCT. (**G**) Levels of LI-secreted cytokines that were significantly increased (*P* < 0.05) in WT recipients as compared with Syn controls after 24 hours of ex vivo culture. (**H**) Levels of serum cytokines that were significantly increased (*P* < 0.05) in WT recipients as compared with Syn controls. Error bars represent standard deviation of the mean of 4 syngeneic controls and 9 allo-HCT recipients. *P < 0.05 as compared with Syn controls. ^#^*P* < 0.05 comparing WT Th and *Gzma*^–/–^ Th recipient mice. n.s., not significant (all by 1-way ANOVA with Tukey’s posttest for multiple comparisons).

CD4^+^ T cells have been previously reported to localize to intestinal crypts and induce aGVHD by destroying cells within this niche (23–25). Consistent with this finding, we observed decreased colon length and increased goblet cell and crypt loss in aGVHD mice as compared with syngeneic controls (Figure 9, A–D; *P* < 0.004). In contrast, *Gzma*^–/–^ Th cell recipient mice exhibited significantly increased colon lengths (*P* = 0.013, Figure 9B) and reduced loss of goblet cells and intestinal crypts as compared with WT controls (Figure 9, A, C, and D; *P* = 0.01 and *P* = 0.02, respectively). This was accompanied by reduced cellular infiltration near crypt bases, as indicated by arrows in Figure 9A. Liver portal inflammation was at similar elevated levels in both WT and *Gzma*-deficient CD4^+^ T cell recipients (*P* = 0.54, Figure 9, A and E), suggesting that CD4^+^ T cell–derived GrA may have a specific role in promoting intestinal aGVHD via destruction of intestinal crypts. Reduced intestinal pathology in *Gzma*^–/–^ Th cell recipient mice did not lead to differences in gut permeability, as measured by serum FITC-dextran levels after oral gavage (Figure 9F) or in intestinal cytokine production (Figure 9G). However, reduced intestinal pathology in *Gzma*^–/–^ recipients did correlate with significantly reduced serum cytokine levels of IL-2, IL-5, and monocyte chemoattractant protein-1 (MCP-1) (*P* = 0.03, *P* = 0.04, *P* = 0.01, respectively) and a decrease in all other serum cytokines that were elevated in WT recipients (*P* > 0.05, Figure 9H).

## Discussion

In this study, we identified a potentially novel subset of GrA-producing CD4^+^ T cells that accumulate specifically within the intestines and were critical for lethal GVHD. From our CyTOF analysis, GrA-producing Th cells were distinct from other Th subsets and did not express high levels of FOXP3 or IL-17A. However, a smaller population of cells exhibited some coexpression of GrB and IFN-γ that segregated independently from TNF/IL-2–coproducing IFN-γ–positive cells. Loss of IFN-γ, TNF, and IL-2 is a hallmark of chronically stimulated (e.g., exhausted) CD4^+^ and CD8^+^ T cells (26, 27). In chronic infections, exhausted Th1 cells take on characteristics of T follicular helper cells (i.e., CXCR5, BCL6, PD-1) and have elevated expression of *Batf*, *Hif1a*, *Maf*, and *Eomes*. It is therefore possible that GrA^+^ Th cells in the intestines might represent a product of chronic Th1 cell activation that has led to reduced IFN-γ production and loss of IL-2 and TNF. In vitro–derived GrA^+^ Th cells also exhibited similar elevated expression of exhausted Th cell genes, including *Il21*, *Maf*, *Batf*, *Hif1a*, and *Eomes* (Figure 4, Supplemental Figure 2, and data not shown). Importantly, while IFN-γ may play a protective role in some aGVHD models, Th cell–derived GrA was clearly detrimental. Therefore, this clearly distinguishes GrA-producing Th cells from IFN-γ–producing Th1 cells in aGVHD.

The cytokines that guide the differentiation of GrA^+^ Th cells in vitro are distinct from other Th lineages. Synergistic signaling between IL-4 and IL-6/21 promoted optimal *Gzma* expression in vitro via alteration of the IL-4–induced gene expression profile in a STAT3- and STAT6-dependent manner. A similar phenomenon was also observed in both mouse and human macrophages polarized with IL-4 only (i.e., M2 macrophages) or in the presence of IL-6 that alters M2-polarized macrophage phenotype and function (28–30). In vivo, both STAT3 and STAT6 were involved in the generation GrA^+^ Th cells during GVHD, with STAT3 having a greater effect. Interestingly, STAT3 and STAT6 had similar roles in maintenance of GrA^+^ Th cell lineage identity as loss of either STAT resulted in increased IFN-γ production. While STAT3 is typically associated with Th17 and T follicular helper cell differentiation (31–34), this pathway is shared by GrA-producing cells. Additionally, loss of STAT3 and STAT6 have each been associated with increased Th1 differentiation and IFN-γ production both in vitro and in vivo (34, 35), indicating that these signaling pathways inhibit Th1 development. Although GrA-producing cells do coexpress IFN-γ, the differential requirement of STAT3 in promoting GrA production and limiting IFN-γ indicates that these cells are divergent from traditional Th1 cells. Further, these data suggest that part of the success of IL-6 blockade therapy for GVHD in mice (36–38) and humans (39, 40) may result from decreasing GrA^+^ Th cells in the intestine.

During aGVHD, the intestines are one of the major target organs of donor Th cells, and their presence in this niche is associated with increased inflammatory cytokine production and lethality (1, 2). In our study, transfer of *Gzma*-deficient CD4^+^ T cells resulted in reduced lethality and reduced goblet cell and intestinal crypt loss as compared with controls. Interestingly, this did not result in changes in intestinal permeability as measured by serum FITC-dextran after oral gavage. These data may indicate that overall barrier permeability is acutely similar, which is mirrored by similar clinical scores at this early time point. At later time points (i.e., >day 15 post-HCT), however, barrier function may be restored more rapidly in the absence of GrA, which may lead to enhanced survival. How GrA^+^ Th cells mediate intestinal damage remains unclear. While GrA was initially shown to cause apoptosis when delivered through perforin-formed membrane pores, GrA was more recently shown to have little cytotoxic potential (41). Additionally, intestinal GrA^+^ Th cells in aGVHD mice did not express perforin (data not shown). GrA has other reported roles in promoting inflammatory cytokine and chemokine production (IL-1β, IL-6, TNF, MCP-1) via interaction with TLRs (42–45). However, these cytokines were not differentially expressed within the intestines, and only MCP-1 was significantly reduced in the serum of *Gzma*^–/–^ recipient mice. Although MCP-1 has documented roles in cellular trafficking during idiopathic pulmonary syndrome (46), we did not observe decreased recruitment of inflammatory cells to the intestines in the absence of GrA. These data indicate that GrA-mediated intestinal damage is likely independent of its role in inflammatory cytokine production and cytolysis.

While Th cells are required in a number of murine models for the development of GVHD, the exact subtypes involved and the effector molecules that they produce to induce disease have been difficult to determine. Treg cells play an important role in limiting intestinal pathology during GVHD. Interestingly, GrA expression is decreased in Treg cells in patients with aGVHD and was required for Treg cell protective function in murine GVHD (22, 47). We observed few FOXP3^+^ Treg cells (<4% of the total CD4s) in the intestines of mice with aGVHD in our studies; however, approximately 20% to 30% of the intestinal Treg cells present did express GrA. While our studies were not designed to study the role of Treg cells in disease, it is possible that GrA in Treg cells and conventional Th cells may have opposite roles in regulating intestinal pathology.

Pathogenic Th subsets have been more difficult to define during aGVHD. Th1-derived IFN-γ has controversial roles in pathogenesis and depends highly on the conditioning regimen (8–10). Likewise, transfer of Th17 cells is sufficient to induce GVHD but with striking specificity for driving pathology in the lung and skin (3). Ullrich et al. (48) described a GM-CSF–producing Th cell subset dependent on basic leucine zipper transcription factor ATF-like (BATF) that was involved in intestinal inflammation. Mechanistically, BATF-deficient cells failed to accumulate within the intestines and induced pathology that is consistent with defects in BATF-deficient cells in inducing colitis (48–50). *Batf* was also highly expressed by GrA^+^ Th cells induced in vitro by IL-4 and IL-6/21, and it is intriguing to speculate that these may be similar cell types. IL-23R^+^CD11c^+^ Th cells were also recently shown to promote intestinal damage during GVHD (51). *Il23r*-deficient donor cells induced reduced liver, lung, and colon pathology scores and dramatically blunted Th1 and Th17 responses, suggesting that IL-23R is required for systemic GVHD effects and may not be specific to intestinal inflammation. In contrast to this report, Buchele et al. suggest that IL-23R on donor T cells may not be required for intestinal GVHD (49). Because IL-23/IL-23R activates STAT3, which in turn can activate BATF in other pathogenic Th cell lineages, it is possible that both IL-23R and BATF are critical regulators of GrA^+^ Th cell differentiation.

In human HCT patients, granzymes have been used as biomarkers of aGVHD and a predictor of disease severity. In an 86-patient cohort, Kircher et al. (52) demonstrated that serum GrA was detected at higher levels than GrB and was a better predictor of disease severity than HLA mismatch or donor/recipient age. Earlier studies by this same group indicated that GrA, but not GrB, produced in mixed lymphocyte reactions between donor and host PBMCs significantly correlated with GVHD grade posttransplant. Further, enhanced GrA expression in these mixed lymphocyte reactions correlated with increased numbers of MHC-II mismatches and outgrowth of CD4^+^ T cells (53). While the cellular source of GrA in actual patients with GVHD remains unclear, these data strongly indicate that CD4^+^ T cells are a potential producer of this protease. Our experiments in a human PBMC-induced NRG mouse model of GVHD support this hypothesis. While GrA was produced by both human CD4^+^ and CD8^+^ T cells in the spleen and liver, human CD4^+^ T cells in the intestines were the dominant producers of GrA and exhibited a noncytolytic phenotype as compared with GrB- and perforin-expressing CD8^+^ T cells in the same tissue. These data combined with the clinical data mentioned above indicate that GrA-producing CD4^+^ T cells may also contribute to human aGVHD. Further, these data indicate that specific GrA inhibitors or broader serine protease inhibitors may represent a novel treatment strategy for patients with GVHD.

## Methods

### Mice and initiation of aGVHD (including clinical scoring).

C57BL/6 and BALB/c mice were purchased from The Jackson Laboratory or Envigo and housed in the animal facility at the Indiana University School of Medicine or within the Purdue University animal facility. *Gzma*^–/–^ mice (54) were housed as above. *Stat6*^–/–^ (55) and C3.SW-H2^b/^SnJ (stock 00438) mice were purchased from The Jackson Laboratory, and *Stat3*^fl/fl^ mice were originally provided by David Levy (New York University, New York, New York, USA) (56) and crossed to CD4-Cre mice to delete STAT3 in T cells. Both male and female mice were used between the ages of 8 and 13 weeks.

In order to initiate aGVHD, BALB/c mice were exposed to 900 cGy of irradiation from a ^137^Cs source or 500 cGy from X-rad 320 x-ray irradiator (Accela). The following day, bone marrow was isolated from the femurs of BALB/c (syngeneic) or C57BL/6 (allogeneic) mice and depleted of T cells using anti–mouse CD90 beads (Miltenyi Biotec), according to manufacturer’s directions, to remove more than 98% of all T cells from the initial population. T cell–depleted (TCD) bone marrow (5 × 10^6^ cells) was injected into irradiated BALB/c recipients along with 10^6^ BALB/c splenocytes (syngeneic) or CD4^+^ (6 × 10^5^) and CD8^+^ T cells (4 × 10^5^) isolated from the spleens of C57BL/6 or *Gzma*^–/–^, *Stat3*^–/–^, or *Stat6*^–/–^ mice (allogeneic) using anti–mouse CD4 or anti–mouse CD8 microbeads (Miltenyi Biotec). The mice were housed in sterilized microisolator cages and were given acidified water (pH < 3) ad libitum beginning the day of irradiation until termination of the experiment. For C3.SW-H2^b/^SnJ recipient mice, male mice were irradiated with 1100 cGy irradiation 24 hours before transfer of 10^7^ TCD bone marrow cells from C57BL/6 donors and 2 × 10^6^ purified T cells (1.2 × 10^6^ CD4^+^, 0.8 × 10^6^ CD8^+^) from WT or *Gzma*^–/–^ donors.

Clinical aGVHD scores were assessed weekly using a previously described scoring system (57). In short, clinical score was measured using the following parameters: weight loss, posture, mobility, skin integrity, stool score, and fur texture. Each parameter was scored 0–2, with 0 being normal and 2 being severe, and added to get a total GVHD clinical score. Mice were euthanized when the clinical score reached 9.0.

### Induction and assessment of the GVL effect.

BALB/c mice were lethally irradiated with 900 cGy on day –1 and received HCT from syngeneic mice (5 × 10^6^ TCD bone marrow cells and 8 × 10^5^ purified T cells) or allogeneic C57BL/6 donors (all received 5 × 10^6^ bone marrow cells and 8 × 10^5^ purified WT T cells, purified T cells from *Gzma*^–/–^ mice, or 5 × 10^5^ purified *Gzma*^–/–^ CD4^+^ T cells and 3 × 10^5^ WT CD8^+^ T cells) along with 4 × 10^5^ GFP^+^ MLL-AF9 cells that have been described elsewhere (58). After HCT and inoculation with leukemia cells, mice were monitored for GVHD-associated clinical score and mortality, and cause of death was determined by accumulative analysis of GVHD score and the frequency of GFP^+^ MLL-AF9 cells in the bone marrow upon euthanization as previously described (58).

### Human PBMC NRG mouse model of GVHD.

To examine GrA production from human CD4^+^ T cells in the context of GVHD, NRG mice (originally from The Jackson Laboratory, purchased from the Purdue Biological Evaluation Core) were injected i.v. with 5 × 10^6^ to 20 × 10^6^ human PBMCs that had been recovered the previous day (37°C, 5% CO_2_, complete RPMI) from frozen stocks isolated from 2 distinct donors (from ZenBio). After injection, mice were monitored daily until they reached 20% body weight loss or developed a significant clinical score (>3). Upon euthanization, immune cells were isolated from the spleen, liver, SI, and LI; were stained for cell surface expression of human CD3, CD4, and CD8; and were fixed, permeabilized, and stained for intracellular human GrA, GrB, and perforin (see below). The frequency of GrA-, GrB-, and perforin-expressing T cells was subsequently determined by flow cytometry.

### Isolation of T cells and dissociation of tissues for cytometry.

Naive CD44^–^CD4^+^ T cells were isolated from the spleen and mesenteric lymph nodes (MesLNs) of C57BL/6 mice via negative magnetic enrichment (Miltenyi Biotec). CD4^+^ and CD8^+^ T cells were isolated from spleens and MesLNs using anti-CD4 and anti-CD8 microbeads following the manufacturer’s instructions (Miltenyi Biotec).

Mononuclear cells were harvested from spleens and MesLNs using sterile frosted glass slides to disrupt the spleen, followed by removal of debris; in some experiments red blood cells were removed with red blood cell lysis buffer (BioLegend). Liver mononuclear cells were isolated by pressing perfused liver tissue through a wire mesh sieve (Bellco Glass) to make a single-cell suspension that was resuspended in 40% Percoll and centrifuged for 10 minutes at 4°C at 872*g* to remove debris and fat/connective tissue. Red blood cell lysis buffer was used to remove residual red blood cells. To isolate mononuclear cells from intestines, small and large intestines were harvested, and Peyer’s patches were removed from small intestinal tissue. Intestines were cut longitudinally and washed with vigorous shaking 3 times with 40 mL of PBS to remove mucus. Washed intestinal tissue was then finely diced with scissors and transferred into digestion medium containing DMEM (Gibco, Thermo Fisher Scientific) with 2% BSA, 10 μg/mL of DNaseI (Roche), and 100 μg/mL of Liberase TL (Roche) or 1.5 mg/mL of type III collagenase (Worthington). Cells were incubated at 37°C for 1.5 hours in a shaking incubator (250 rpm) before straining through a 100 μm nylon sieve and washing with PBS containing 2% fetal calf serum. The resulting cells were resuspended in 40% Percoll and centrifuged as with liver-derived samples per above. Viable cells isolated from each tissue were enumerated via trypan blue staining and counted on a hemocytometer.

### Cell stimulation, flow cytometry, and CyTOF.

Antibodies used for flow cytometry are listed in Supplemental Table 1. Briefly, cells (1 × 10^6^ to 2 × 10^6^) isolated from various tissues were left unstimulated (complete media only) or stimulated in complete media supplemented with PMA (50 ng/mL) and ionomycin (1 μg/mL) in the presence of 2 μM monensin (MilliporeSigma) for 5 hours at 37°C. Following stimulation, cells were preincubated with purified anti-CD16/32 antibodies and fixable viability dye (eBioscience, Thermo Fisher Scientific) and subsequently stained for surface markers (i.e., CD3, CD4). Cells were washed with FACS buffer (PBS, 0.1% BSA, 0.01% sodium azide) and fixed with 3.7% formaldehyde (*v/v* in PBS) for 20 minutes at 4°C. Cells were subsequently washed and permeabilized in 1× permeabilization buffer (eBioscience, Thermo Fisher Scientific) and stained for intracellular cytokines and granzymes.

For CyTOF experiments, cells were stimulated and fixed as above; however, antibody reagents were developed specifically for use with CyTOF and purchased from Fluidigm unless otherwise noted (Supplemental Table 2). After incubation, cells were washed with PBS (Rockland Immunochemicals Inc.), and their viability was assessed by cisplatin (Fluidigm Inc.). Cells were then washed with Maxpar cell staining buffer (Fluidigm Inc.). Cells were fixed in PBS with 1.35% formaldehyde (MilliporeSigma) for 10 minutes at room temperature. Samples to be barcoded by Pd isotope were resuspended in barcode perm buffer (Fluidigm Inc.). Appropriate barcodes in barcode perm buffer were transferred into each individual sample and incubated for 30 minutes at room temperature. After washing with Maxpar cell staining buffer, all barcoded samples were combined into 1 tube. To prevent high nonspecific background signal, Fc blocking (BioLegend) was performed, followed by surface antibody cocktail staining. After permeabilization using the Nuclear Antigen Staining Buffer Set (Fluidigm Inc.), cells were stained with intracellular and transcription factors antibody cocktail. Cells were then stained with ^193^Ir DNA-intercalator in Maxpar fix and perm buffer (Fluidigm Inc.) to identify all nucleated cells. In the final step, cells were washed by type I, ultrapure water (18.2 MΩ) and filtered into cell strainer cap tubes (40 μm). Prior to the data acquisition, cell concentration was adjusted to 5 × 10^5^/mL to obtain approximately 500 events/s. To normalize data, cells were diluted in ultrapure water containing 0.1x EQ Four Element Calibration Beads (Fluidigm Inc.). Samples were then injected into cyTOF2 mass cytometer machine (Fluidigm Inc.). Data were acquired from barcode channels (channels 102, 104, 105, 106, 108, and 110), EQ beads channels (channels 140, 151, 153, 165, and 175), Cisplatin channel (channel 195), cell intercalator ^193^Ir, and corresponding channels to the antibody panel (Supplemental Table 2).

### In vitro culture of Th cells.

Naive CD4^+^ T cells were cultured in tissue culture–treated multiwell plates that were coated overnight with 2 μg/mL of anti-CD3ε (Bio X Cell) at 10^6^ cells/mL. Th1, Th2, and Th9 were cultured as previously described (59), and in some cases IL-6 and/or IL-21 was added to these cultures in varying concentrations as noted. In cytokine screening experiments, cells were cultured in flat-bottom, 96-well plates with plate-bound anti-CD3 and soluble anti-CD28 as per above. Cytokines were added at day 0 of culture (IL-4, 10 ng/mL; IL-6, 20 ng/mL; IL-12, 10 ng/mL; IL-21, 100 ng/mL; Pam3CSK4, 2 μg/mL; TGF-β, 2 ng/mL; TNF 100 ng/mL; see Supplemental Table 3 for other cytokines used), and cells were expanded 1:4 in fresh media with no additional cytokines day 3 of culture. All cytokines were purchased from PeproTech, except TGF-β, which was purchased from R&D Systems, Bio-Techne. Pam3CSK4 was purchased from InvivoGen.

### Serum/intestinal cytokine analysis, permeability, and histology.

Serum was collected by cardiac puncture immediately after CO_2_ asphyxiation and allowed to clot for 6 hours at 4°C. After clotting, samples were centrifuged for 10 minutes at 2000*g*, and serum was aliquoted into fresh tubes. For intestinal cytokine analysis, 2 cm sections of midcolon were collected into 1 mL of sterile complete RPMI medium and incubated for 24 hours at 37°C and 5% CO_2_. Medium was collected and centrifuged for 10 minutes at 2000*g* to remove debris, and clarified medium was frozen at –20°C until cytokine measurements. Cytokine levels were assessed by mouse cytokine Bio-Plex Pro (23-plex, Bio-Rad) following the manufacturer’s directions at the Multiplex Analysis Core within the Indiana University Melvin and Bren Simon Cancer Center.

Intestinal permeability was measured by FITC-dextran permeability assays. In these studies, BALB/c mice received bone marrow and T cells from BALB/c (syngeneic) or WT or *Gzma*^–/–^ mice (allogeneic) as above. At day 9 post-HCT, mice were orally gavaged with 150 μL of 80 mg/mL FITC-dextran (4 kDa, MilliporeSigma) dissolved in PBS, and 4 hours later blood was harvested by retro-orbital bleed; serum was prepared as above. Serum samples were immediately assayed for FITC fluorescence signal via a fluorescent plate reader (Synergy 4, BioTek Instruments).

For histology, liver and LI tissue were stored in buffered formalin (Formal-Fixx, Thermo Fisher Scientific), and tissue sectioning, H&E staining, and imaging were performed by HistoWiz (https://home.histowiz.com). Histological analysis was done blinded and intestines were scored according to current guidelines (60) whereas portal inflammation was scored via the Knodell scale (61).

### RNA extraction, real-time PCR, and RNA-Seq analysis.

RNA was extracted from 5 mm sections of SI or colon and placed directly in 1 mL of Trizol (Ambion) and snap-frozen at –80°C. After thawing, tissues were homogenized for 30 seconds or until tissue was completely dissociated. RNA from in vitro Th cultures was harvested by placing 2 × 10^5^ to 2 × 10^6^ Th cells in Trizol reagent. RNA was isolated from these preparations via phenol-chloroform extraction and assayed via real-time PCR as previously described (17).

For RNA-Seq, cells were isolated in Trizol reagent and RNA was isolated as above. Illumina-sequenced reads from these cells were further quality filtered, aligned to the mouse genome (mm10), postprocessed, quantified, and analyzed by Otogenetics. The quantified expression profile for each condition was further examined for downstream analysis. Test of significance was performed using CuffDiff (62) (and adjusted to *q* < 0.05) between each pair of conditions (i.e., Th0 vs. Th0+IL-6/21, Th0 vs. Th0+IL-4, and Th0 vs. Th0+IL-4+IL-6/21). Differentially expressed genes (at 5% FDR) were represented as 2-fold upregulated and downregulated genes. RNA-Seq data were deposited into the National Center for Biotechnology Information’s Gene Expression Omnibus database (GSE154660).

### Cytometry data analysis.

For mass cytometry files, the concatenated normalized fcs files were uploaded to a Cytobank web server (Cytobank Inc.) for gating out dead cells and quality control. To run further analysis on multiplexed samples, the debarcoding protocol (Fluidigm Inc.) was performed with the Barcode Separation 0.16 and Mahalanobis Distance 5 values. viSNE analyses were performed on cytometry data from all samples. To be more informative in visualizing each cluster on the viSNE plot, we ran SPADE on viSNE and overlaid different populations on a 2D tSNE plot. Flow cytometry data were analyzed with FlowJo (v8.9, Tree Star).

### Statistics.

Standard data plots and statistics (2-tailed Student’s *t* test or 1-way ANOVA with Tukey’s posttest, indicated in figure legend text) were produced with Prism (GraphPad Prism, v8) and were considered significant if *P* < 0.05. Survival plots were analyzed for significance by a log-rank test, where differences between 2 groups were assessed by using 2-tailed unpaired Student’s *t* tests or Mann-Whitney *U* test. Bonferroni’s correction was used when comparing multiple groups. All statistical tests were done with GraphPad Prism v8.

### Study approval.

All mice used and mouse studies were with the approval of the Indiana University or Purdue University IACUC.

## Author contributions

S Park, BG, JA, HJ, PMB, SC, TI, and TAH performed experiments and analyzed data from experiments. MK, RS, and SCJ analyzed RNA-Seq data and aided in the interpretation of RNA-Seq results. JP provided the *Gzma*^–/–^ mice and provided insight on the manuscript. S Paczesny provided insight on the experimental design for the GVHD and GVL experiments and helped write the manuscript. MHK provided insight on the experimental design of Th cell culturing experiments and data analysis and aided in writing of the manuscript. MRO performed experiments, analyzed data, and wrote the manuscript.

## Supplementary Material

Supplemental data

## Version 1 08/18/2020

In-Press Preview

## Version 2 09/17/2020

Electronic publication
